# Age-Based Screening for Lung Cancer Surveillance in the US

**DOI:** 10.1001/jamanetworkopen.2025.46222

**Published:** 2025-11-20

**Authors:** Hee Chul Yang, Austin Chang, Maxime Visa, Andrew Yoon, Alexandra Abbott, Yangruijue Ma, Mohamed E. Abazeed, Eric M. Hart, Bradley D. Allen, John E. Pandolfino, Yu-Wei Liu, Dean P. Schraufnagel, Christopher Kapp, Sean Smith, Samuel Kim, Kalvin Lung, Momen M. Wahidi, G. R. Scott Budinger, Masha Kocherginsky, Trevor Barnum, Ankit Bharat

**Affiliations:** 1 Division of Thoracic Surgery, Canning Thoracic Institute, Northwestern University Feinberg School of Medicine, Chicago, Illinois; 2 Department of Preventative Medicine, Northwestern University Feinberg School of Medicine, Chicago, Illinois; 3 Department of Radiation Oncology, Northwestern University Feinberg School of Medicine, Chicago, Illinois; 4 Department of Radiology, Northwestern University Feinberg School of Medicine, Chicago, Illinois; 5 Division of Gastroenterology and Hepatology, Northwestern University Feinberg School of Medicine, Chicago, Illinois; 6 Division of Thoracic Surgery, Department of Surgery, Kaohsiung Medical University Hospital, Kaohsiung Medical University, Kaohsiung, Taiwan; 7 Division of Pulmonary and Critical Care Medicine, Northwestern University Feinberg School of Medicine, Chicago, Illinois

## Abstract

**Importance:**

Lung cancer is increasingly diagnosed in never-smokers, suggesting that the current US Preventive Services Task Force (USPSTF) screening criteria using low-dose computed tomography exclude many at-risk individuals, thus raising concerns about their effectiveness and equity.

**Objective:**

To evaluate the proportion, clinical characteristics, and survival of patients with lung cancer who meet or are excluded from USPSTF criteria and to use institutional and published data to model expanded criteria and evaluate age-based screening.

**Design, Setting, and Participants:**

Patients with lung cancer diagnosed from 2018 to 2023 at an academic center were analyzed, with follow-up through 2024. Patients were stratified by 2021 USPSTF eligibility (age 50-80 years, ≥20 pack-years, and current or quit <15 years). Expanded scenarios were modeled and benchmarked against breast and colorectal screening.

**Exposure:**

Diagnosis of lung cancer.

**Main Outcomes and Measures:**

The primary outcome was the proportion of patients meeting USPSTF criteria. Secondary outcomes included survival, clinical characteristics, and modeled impact of expanded screening scenarios on detection, cost-effectiveness, and risks. Group comparisons were performed using Pearson χ^2^ or Fisher exact tests for categorical variables and Wilcoxon rank-sum tests for continuous variables. Cox proportional hazards models were used to calculate hazard ratios (HRs) with 95% CIs.

**Results:**

Among 997 patients (median [IQR] age, 67 [18-99] years; 577 [58.0%] women), only 350 (35.1%) met USPSTF criteria. The nonguideline group included more women (396 of 647 patients [61.0%] vs 181 of 350 patients [52.0%]), Asian patients (62 of 647 patients [9.6%] vs 13 of 350 patients [3.7%]), and never-smokers (247 of 647 patients [38.0%]); had more adenocarcinoma diagnoses (469 of 647 patients [72.0%] vs 192 of 350 patients [55.0%]); and had better overall survival (median [IQR], 9.5 [6.6-12.3] vs 4.4 [3.7-6.0] years; HR, 0.67; 95% CI, 0.55-0.82; *P* < .001) than the guideline group. Never-smokers (247 of 997 patients [24.8%]), those who quit longer than 15 years (134 of 997 patients [13.0%]), those with less than 20 pack-years (65 of 997 patients [6.5%]), and those aged outside the 50 to 80 year range (41 of 997 patients [4.1%]) were excluded. Expanding criteria to age 40 to 85 years, 10 or more pack-years, and no cessation limit increased detection to 62.1% (619 of 997 patients). A modeled age-based screening (40-85 years) captured 93.9% of cases (936 of 997 patients) and prevented 26 124 deaths annually (95% CI, 20 000-32 248 deaths annually) at $101 000 per life saved (95% CI, $82 000-$120 000), compared with $890 000 (95% CI, $700 000-$1 100 000) for breast cancer and $920 000 (95% CI, $700 000-$1 200 000) for colorectal cancer screening. Sensitivity analysis confirmed robust findings across all parameter ranges, with 98.7% probability of superior cost-effectiveness.

**Conclusions and Relevance:**

Current USPSTF guidelines exclude two-thirds of patients, disproportionately women and never-smokers. Age-based screening substantially improved detection, demonstrated 6-fold superior cost-effectiveness compared with existing programs, and addressed inequities.

## Key Points

**Question:**

What proportion of patients with lung cancer meet US Preventive Services Task Force (USPSTF) screening criteria, and would age-based screening improve detection and be cost-effective?

**Findings:**

In a cohort of 997 patients with lung cancer, only 35.1% met USPSTF criteria; noneligible patients had better survival. Expanding criteria to ages 40 to 85 years and 10 or more pack-years increased the detection rate to only 62.1%, whereas universal age-based screening (40-85 years) detected 93.9% of cancers, preventing at least 26 124 deaths annually at $101 000 per life saved vs $890 000 for breast and $920 000 for colorectal screening.

**Meaning:**

These findings suggest that current screening guidelines miss most patients with lung cancer, and age-based screening could improve detection and cost-effectiveness while reducing disparities.

## Teaser

This cohort study examines the proportion, clinical characteristics, and survival of patients with lung cancer who meet or are excluded from the current US Preventive Services Task Force criteria.

## Introduction

Lung cancer is the leading cause of cancer-related mortality worldwide, surpassing the annual deaths from breast, prostate, and colorectal cancers.^1^ Despite advances in therapies, 5-year survival rates remain at 21%, because 75% of patients receive a diagnosis at advanced stages.^2^ Low-dose computed tomography (LDCT)–based screening of patients deemed to be at high risk for lung cancer on the basis of tobacco-smoking exposure has proven effective for early detection, with the National Lung Screening Trial demonstrating a 20% reduction in lung cancer mortality.^3^ This prompted the US Preventive Services Task Force (USPSTF) to recommend annual screening for adults aged 50 to 80 years with at least 20 pack-year smoking history who currently smoke or quit within 15 years.^4^

Lung cancer screening faces critical implementation challenges. Participation rates remain below 15% of currently eligible individuals,^5^ contrasting sharply with 67% to 69% participation rates for breast and colorectal cancer screening, which increased after their transition from risk-based to age-based guidelines.^6,7^ Multiple nontobacco risk factors are established in lung cancer pathogenesis, including environmental exposures (radon, air pollution, and asbestos), occupational hazards, genetic predispositions, and chronic pulmonary diseases.^8,9^ Indeed, radon is the second leading cause of lung cancer in the US,^10^ while fine particulate matter (ie, particles that are ≤2.5 µm in diameter) pollution increases lung cancer risk in both smokers and nonsmokers.^11,12^ Furthermore, risks from e-cigarette and marijuana exposure remain uncertain. Hence, eligibility criteria based solely on tobacco use exclude growing at-risk populations of never, limited, or passive tobacco smokers who develop lung cancer.^13^

Exclusive reliance on tobacco smoking for screening eligibility also raises health equity concerns. Socioeconomically disadvantaged individuals and those from racial and ethnic minoritized groups disproportionately experience secondhand smoke, elevated pollution, and radon exposure.^14,15^ This might explain why African American individuals develop lung cancer with fewer reported pack-years.^16,17^ Women and certain ethnic groups are also at increased risk for developing lung cancer with minimal tobacco smoking history.^13,18^ Furthermore, self-reported quantification of lifetime cigarette smoke exposure using pack-years is inaccurate, with studies demonstrating a 40% discrepancy between reported and actual smoking histories.^19^

Although recent data suggest that early detection in the general population can improve outcomes,^20^ the efficacy of USPSTF lung cancer screening guidelines and the benefits of a non–risk-based approach remains unclear. Our study addresses this gap by evaluating characteristics and survival outcomes of patients with lung cancer who met or were excluded from USPSTF guidelines, modeling age-based lung cancer screening criteria to improve detection, survival, cost-effectiveness, and equity.

## Methods

### Institutional Study Cohort

Detailed methods are shown in the eAppendix in Supplement 1. We first identified consecutive patients, retrospectively, with lung cancer starting March 20, 2023, and selected sequentially backward until September 2018 with study follow-up until December 2024. Patients were stratified by 2021 USPSTF screening criteria into guideline (age 50-80 years, ≥20 pack-years, current smoking, or quit <15 years) and nonguideline groups. This report was prepared in accordance with Strengthening the Reporting of Observational Studies in Epidemiology (STROBE) reporting guideline. All patient data were deidentified and handled in accordance with the Declaration of Helsinki.^21^ The study was approved by the institutional review board at Northwestern University, with a waiver for informed consent.

Data were extracted from electronic medical records (EMRs) and included demographics, smoking history, clinical presentation, referral source, imaging indication, pulmonary function tests, tumor characteristics, and survival outcomes. Race and ethnicity were recorded in the EMR on the basis of patient self-report at the time of clinical registration. Categories were defined by the EMR system as Asian, Black or African American, Hispanic or Latino, White, and other, which includes individuals reporting multiracial identity, American Indian or Alaska Native, Native Hawaiian or other Pacific Islander, or those who declined to answer. Data on race and ethnicity are included to identify potential racial and ethnic disparities in lung cancer screening participation and outcomes. Pack-year smoking history was extracted from structured EMR fields. Survival status was ascertained through the institutional EMR, which is integrated through the care-everywhere module allowing access to medical information provided at other institutions and supplemented with linkage to the Social Security Death Index to ensure completeness of mortality follow-up. For all patients with lung cancer diagnosed at our institution, external chest CT imaging, including any prior LDCT scans performed elsewhere, was systematically uploaded into the EMR. This process ensured that LDCT screening data were comprehensively captured, irrespective of the site of imaging.

### Statistical Analysis

Descriptive statistics were summarized as means with SDs or medians with IQRs for continuous variables and as counts with percentages for categorical variables. Group comparisons were performed using Pearson χ^2^ or Fisher exact tests for categorical variables and Wilcoxon rank-sum tests for continuous variables. Survival was estimated using Kaplan-Meier methods and compared with log-rank tests. Cox proportional hazards models were used to calculate hazard ratios (HRs) with 95% CIs. All *P* values were 2-sided, with *P* < .05 considered statistically significant.

To assess cost-effectiveness, we developed Monte Carlo simulations (10 000 iterations) that modeled program participation, detection rates, costs, and outcomes, and generated 95% CIs using appropriate probability distributions for each parameter. Analyses were conducted at observed uptake (14.4%) and a hypothetical 70% participation rate, comparable to national breast (67%) and colorectal (69%) screening. Incremental cost-effectiveness ratios (ICERs) were expressed in 2023 US dollars per life saved and per quality-adjusted life-year (QALY). Radiation-induced cancers were estimated using BEIR VII models, incorporating sex, age, latency, and competing mortality. A sensitivity analysis incorporated a 6.7% all-cause mortality reduction, as reported in prior LDCT trials.

To evaluate mortality benefits from earlier detection, we applied stage-shift models using our institutional cohort. The number needed to screen was calculated as the reciprocal of the absolute risk reduction in mortality. We compared lung cancer screening outcomes with established breast (biennial, ages 40-74 years) and colorectal (ages 45-75 years) screening programs. Parity with breast and colorectal national screening was defined as achieving their respective modeled annual numbers of lives saved (10 660 for breast and 13 650 for colorectal) extracted from Surveillance, Epidemiology, and End Results estimates.^22^ Modeling scenarios also tested incremental expansions of lung cancer screening eligibility criteria, including extending the age range to 40 to 85 years, lowering the smoking threshold to 10 pack-years, eliminating the 15-year cessation limit, and combinations of these modifications. Age-based screening (ages 40-85 years) was simulated to estimate annual lung cancer deaths prevented. Sensitivity analyses examined parameter uncertainty through one-way variation of key inputs and scenario analyses combining multiple parameter changes. All statistical analyses were conducted in R statistical software version 4.3.1 (R Project for Statistical Computing) using the survival, gtsummary, and ggplot2 packages. Two independent biostatisticians (Y.M. and M.K.) verified the analyses.

## Results

### Study Cohort Characteristics

Among 997 patients with lung cancer (median [IQR] age, 67 [18-99] years; 577 [58.0%] women; 75 [7.5%] Asian, 160 [16.0%] Black, 41 [4.1%] Hispanic, 674 [68.0%] White, and 70 [7.0%] other races), only 350 (35.1%) met USPSTF screening criteria (guideline group), and 647 (64.9%) did not (nonguideline group). The nonguideline group differed significantly from the guideline group in several characteristics. Patients were younger (median [IQR] age, 66 [50-80] vs 69 [18-99] years), with a broader age distribution (18-99 years) reflecting inclusion of patients outside the USPSTF-defined age range of 50 to 80 years. Women comprised a larger proportion of the nonguideline group vs the guideline group (396 of 647 patients [61.0%] vs 181 of 350 patients [52.0%]), as did Asian patients (62 of 647 patients [9.6%] vs 13 of 350 patients [3.7%]), whereas other racial groups showed similar distributions (Table 1).

**Table 1.** Baseline Demographic and Clinical Characteristics of Patients With Lung Cancer Stratified by US Preventive Services Task Force Lung Cancer Screening Eligibility

Characteristic	Patients, No. (%)			*P* value^a^
	Overall (N = 997)	Guideline (n = 350)	Nonguideline (n = 647)	
Age at diagnosis, median (range), y	67 (18-99)	66 (50-80)	69 (18-99)	.003
Sex				
Female	577 (58.0)	181 (52.0)	396 (61.0)	.004
Male	420 (42.0)	169 (48.0)	251 (39.0)	
Race				
Asian	75 (7.5)	13 (3.7)	62 (9.6)	<.001
Black or African American	160 (16.0)	63 (18.0)	97 (15.0)	.20
White	674 (68.0)	243 (69.0)	431 (67.0)	.40
Other^b^	70 (7.0)	22 (6.3)	48 (7.4)	.50
Ethnicity				
Hispanic or Latino	41 (4.1)	19 (5.4)	22 (3.4)	.20
Not Hispanic, Latino, or Spanish origin	886 (89.0)	311 (89.0)	575 (89.0)	
Patient declined to answer	70 (7.0)	20 (5.7)	50 (7.7)	
Smoking status at diagnosis				
Never	247 (25.0)	0	247 (38.0)	<.001
Current	166 (17.0)	138 (39.0)	28 (4.3)	
Former	584 (59.0)	212 (61.0)	372 (57.0)	
Pack-years, median (IQR)	30 (18-50)	40 (30-55)	20 (8-35)	<.001
Unknown	263	0	263	
Quit time to diagnosis, mean (SD), y	14 (16)	3 (5)	24 (16)	<.001
Unknown	248	0	248	
Pretreatment forced expiratory volume in 1 second, mean (SD), %	80 (45)	70 (20)	86 (55)	<.001
Unknown	495	153	342	
Pretreatment diffusing capacity of the lung for carbon monoxide, mean (SD), %	68 (20)	64 (19)	71 (20)	<.001
Unknown	605	199	406	
Previous malignant cancer				
None	765 (77.0)	284 (81.0)	481 (74.0)	.02
Yes	232 (23.0)	66 (19.0)	166 (26.0)	
Low-dose computed tomography Lung Rads Category				
2	6 (13.0)	6 (14.0)	0	>.90
2S	1 (2.2)	1 (2.3)	0	
4A	7 (16.0)	7 (16.0)	0	
4B	6 (13.0)	6 (14.0)	0	
4X	25 (56.0)	24 (55.0)	1 (100.0)	
Unknown	952	306	646	
Coronary artery disease				
No	614 (62.0)	203 (58.0)	411 (64.0)	.09
Yes	383 (38.0)	147 (42.0)	236 (36.0)	
P stage				
I	251 (25.0)	72 (21.0)	179 (28.0)	.02
II	115 (12.0)	43 (12.0)	72 (11.0)	
III	191 (19.0)	82 (24.0)	109 (17.0)	
IV	433 (44.0)	150 (43.0)	283 (44.0)	
Unknown	7	3	4	
Histologic profile				
Adenocarcinoma	661 (66.0)	192 (55.0)	469 (72.0)	<.001
Carcinoid	29 (2.9)	2 (0.6)	27 (4.2)	.001
Large cell	14 (1.4)	4 (1.1)	10 (1.5)	.80
Non–small cell lung cancer, not otherwise specified	73 (7.3)	31 (8.9)	42 (6.5)	.20
Sarcomatoid	3 (0.3)	1 (0.3)	2 (0.3)	>.90
Small cell lung cancer	70 (7.0)	46 (13.0)	24 (3.7)	<.001
Squamous cell carcinoma	146 (15.0)	73 (21.0)	73 (11.0)	<.001

^a^ *P* values were calculated with Wilcoxon rank sum test, Pearson χ^2^ test, or Fisher exact test.

^b^ The other category included individuals reporting multiracial identity, American Indian or Alaska Native, Native Hawaiian or other Pacific Islander, or those who declined to answer.

Smoking histories differed markedly between groups. Although all guideline group patients were smokers, the nonguideline group included 247 (38.0%) never-smokers and 372 (57.0%) former smokers who quit more than 15 years prior or had less than 20 pack-years of smoking history. Pack-year exposure was lower in the nonguideline group vs the guideline group (median [IQR], 20 [8-35] years vs 40 [30-55] years), with longer cessation duration (mean [SD], 24 [16] years vs 3 [5] years). Histologic distribution and clinical characteristics also varied significantly. Adenocarcinoma predominated in the nonguideline group (469 of 647 patients [72.0%] vs 192 of 350 patients [55.0%]), whereas squamous cell carcinoma (74 of 350 patients [21.0%] vs 71 of 647 patients [11.0%]) and small cell lung cancer (46 of 350 patients [13.0%] vs 24 of 647 patients [3.7%]) were more common in the guideline group. Despite similar rates of stage IV disease, the nonguideline group had more stage I diagnoses (179 of 647 patients [28.0%] vs 72 of 350 patients [21.0%]). Pulmonary function was better preserved in the nonguideline group (mean [SD] pretreatment forced expiratory volume in 1 second, 86% [55%] vs 70% [20%] estimated; pretreatment diffusing capacity of the lung for carbon monoxide mean [SD], 71% [20%] vs 64% [19%]), consistent with their lower smoking burden. Notably, prior malignant cancers were more frequent in the nonguideline group (168 of 647 patients [26.0%] vs 67 of 350 patients [19.0%]). Emergency department presentations accounted for approximately 32% of diagnoses in both groups (210 of 647 patients vs 110 of 350 patients), often with acute symptoms suggesting advanced disease. Primary care practitioners referred approximately 28% of patients, typically after abnormal chest x-rays or symptom evaluation. Respiratory specialists referred more guideline group patients (28 of 350 patients [8.0%] vs 31 of 647 patients [4.8%]), whereas thoracic surgery referrals were similar between groups (5 of 350 patients [1.4%] vs 22 of 647 patients [3.4%]) (eTable 1 in Supplement 1).

### Survival Outcomes

Median overall survival (OS) for the entire cohort was 6.56 years (95% CI, 5.43-7.52 years), with 5-year survival rates of 54.6% (544 of 997 patients) (Figure, panel A). Compared with current smokers, never-smokers had reduced mortality (HR, 0.49; 95% CI, 0.35-0.67; *P* < .001). Among former smokers, cessation duration was associated with improved survival: recent quitters (≤10 years) showed no benefit (HR, 0.92; 95% CI, 0.70-1.22; *P* = .60), whereas those who quit 10 to 30 years prior (HR, 0.73; 95% CI, 0.54-0.99; *P* = .05) and more than 30 years prior (HR, 0.65; 95% CI, 0.46-0.92; *P* = .02) had progressively lower mortality (eTable 2 in Supplement 1).

**Figure.**
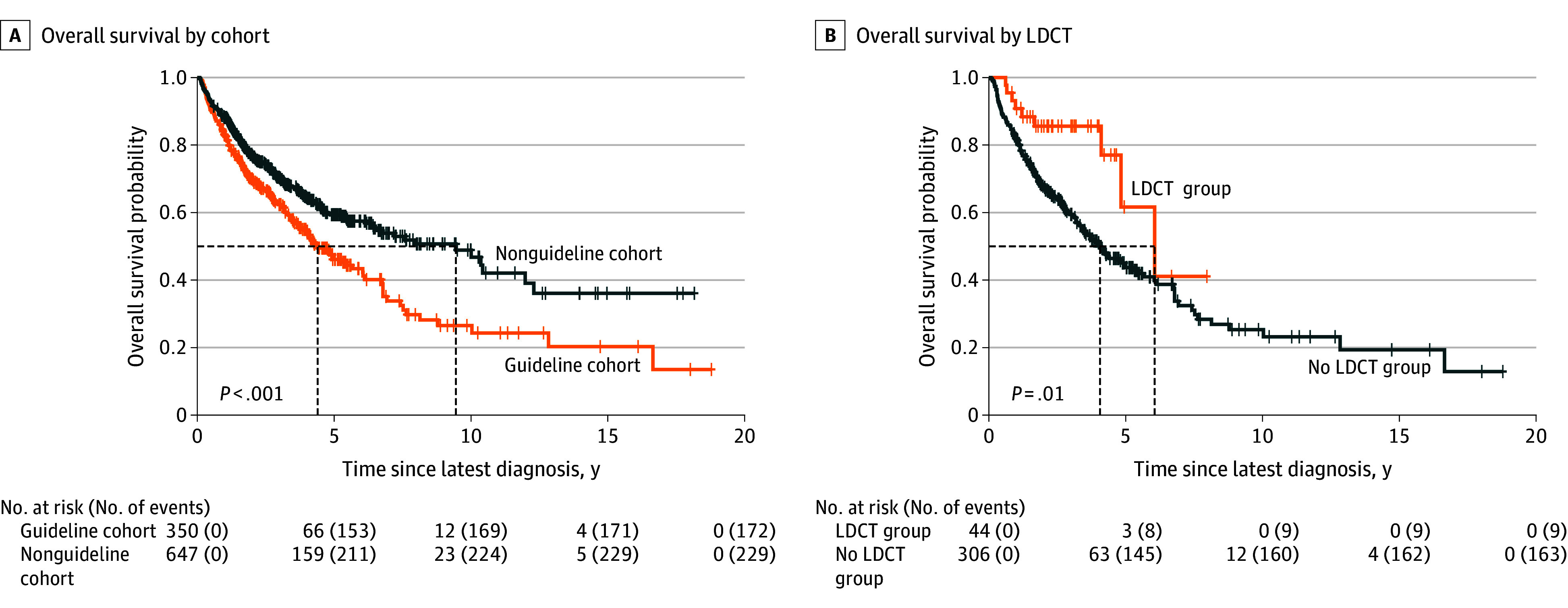
Survival Analyses of Patients With Lung Cancer by Screening Eligibility and Detection Method Panel A shows overall survival by US Preventative Services Task Force eligibility (guideline vs nonguideline). Panel B shows overall survival for patients whose cancer was diagnosed by low-dose computed tomography (LDCT) vs no LDCT in the guideline group.

The nonguideline group had higher probability of survival compared with the guideline group (HR, 0.67; 95% CI, 0.55-0.82; *P* < .001), with better median survival (9.5 [6.6-12.3] vs 4.4 [3.7-6.0] years) and OS at all time points (5-year, 59.3% [384 of 647 patients] vs 46.1% [161 of 350 patients]; 10-year, 48.9% [317 of 647 patients] vs 26.5% [93 of 350 patients]; and 15-year, 36.1% [234 of 647 patients] vs 20.3% [71 of 350 patients]) (Figure, panel A). Other poor prognostic factors included male sex (HR, 1.57; 95% CI, 1.29-1.91; *P* < .001) and advanced stage, with HRs of 2.06 (95% CI, 1.32-3.22; *P* < .001) for stage 2, 2.88 (95% CI, 1.99-4.18; *P* < .001) for stage 3, and 5.97 (95% CI, 4.35-8.20; *P* < .001) for stage 4. Among histologic subtypes, carcinoid tumors had the best prognosis (HR, 0.20; 95% CI, 0.06-0.61; *P* = .005), whereas small cell lung cancer carried the worst prognosis (HR, 2.94; 95% CI, 2.17-4.00; *P* < .001) (eTable 3 in Supplement 1).

Patients who underwent LDCT screening (45 patients; 12.6% of guideline patients underwent LDCT screening) and received a diagnosis of lung cancer had better 5-year OS (58.9%; 95% CI, 35.4%-98.0%) than the 952 patients who did not receive screening (54.2%; 95% CI, 50.5%-58.1%), although the difference was not statistically significant (*P* = .10). Within the guideline group specifically, patients whose diagnosis was made by LDCT had earlier stage disease (eTable 4 in Supplement 1) and improved survival (Figure, panel B). Approximately 25% of initial LDCT scans identified nodules requiring follow-up.

### Reasons for Screening Ineligibility and Evaluation of Expanded Screening

Analysis of the 647 patients in the nonguideline group revealed multiple exclusion factors (Table 2). Age alone excluded 41 patients (4.1%), including 9 patients younger than 50 years and 32 patients older than 80 years. Smoking-related exclusions included less than 20 pack-years alone (65 of 997 patients [6.5%]), quit more than 15 years prior despite heavy smoking (134 of 997 patients [13.4%]), and both factors combined (89 of 997 patients [8.9%]). Never-smokers represented 247 of 647 patients (38.0%) in the nonguideline cohort and 247 of 997 patients (24.8%) overall, highlighting the substantial proportion of lung cancers unrelated to tobacco exposure. Accordingly, we found incremental expansions that would improve detection: extending age to 40 to 85 years (additional 33 of 997 patients [3.3%]), lowering the threshold to 10 pack-years (41 of 997 patients [4.1%]), eliminating the 15-year cessation limit (130 of 997 patients [13.0%]), and combining all 3 (58 of 997 patients [5.8%]). These modifications would increase detection to 619 of 997 patients (62.1%), capturing an additional 269 patients (27.0%). However, 247 of 647 patients (38.0%) in the nonguideline group, including all never-smokers, would remain ineligible. In contrast, a non–risk-based, age-based approach (40-85 years) captured 936 of 997 patients (93.9%) (eTable 5 in Supplement 1).

**Table 2.** Reasons for Screening Ineligibility Among Patients in the Nonguideline Group

Study cohort	Patients, No. (%) (N = 997)
Eligible	
Age 50-80, y	
≥20 PY and quit <15 y	350 (35.1)
Ineligible	647 (64.9)
1 Criterion	
Not in age range	41 (4.1)
Quit >15 y	134 (13.4)
<20 PY	65 (6.5)
2 Criteria	
Not in age range and quit >15 y	32 (3.2)
Not in age range and <20 PY	15 (1.5)
<20 PY and quit >15 y (smoker)	89 (8.9)
Age-eligible never-smokers	195 (20)
All criteria	76 (7.6)

Abbreviation: PY, pack-years.

### Mortality Impact and Stage-Shift Requirements of Age-Based Lung Cancer Screening

Informed by our institutional data and similar to breast and colon cancer screening approaches, we determined the efficacy of a non–risk-based, age-based (ages 40-85 years) LDCT screening program. We found that the number needed to screen to prevent 1 lung cancer death was approximately 644 individuals (95% CI, 314-1100 individuals), compared with 3037 individuals (95% CI, 1774-4891 individuals) for breast cancer (ages 40-74, biennial) and 766 individuals (95% CI, 394-1318 individuals) for colorectal cancer (ages 45-75 years). To achieve parity with breast cancer screening (10 660 lives saved annually), age-based lung cancer screening would need to detect 21.4% of lung cancer at stage I, whereas matching colorectal cancer screening (13 650 lives saved) would require 22.3% stage I detection, an increase of just 5.4% and 6.3%, respectively, over the 16.0% rate found in the guideline group (Table 3). Indeed, our data showing 28.0% stage I detection in nonguideline patients suggested these targets were achievable. At a 30% stage I detection rate, universal screening would prevent 26 124 deaths annually (95% CI, 20 000-32 248 deaths annually), exceeding combined breast and colorectal screening programs (24 310 deaths prevented) (Table 3). Sensitivity analyses confirmed robust findings across all parameter ranges (eTable 6 in Supplement 1).

**Table 3.** Stage-Shift Scenarios and Projected Outcomes

Scenario	Stage I detection rate, %	Mean (95% CI)^a^		
		Lives saved annually	Cost per life saved$	NNS to prevent 1 death
Current lung screening (USPSTF guidelines)	16.0	3650 (2800-4500)	219 000 (178 000-260 000)	222 (180-274)
Match breast screening	21.4	10 660 (8500-12 820)	248 000 (206 000-290 000)	238 (198-286)
Match colorectal screening	22.3	13 650 (11 000-16 300)	194 000 (163 000-225 000)	238 (200-284)
Realistic projection	30.0	26 124 (20 000-32 248)	101 000 (82 000-120 000)	238 (193-293)
Optimal projection	35.0	33 461 (26 000-40 922)	79 000 (65 000-93 000)	238 (195-290)

Abbreviations: NNS, number needed to screen; USPSTF, US Preventive Services Task Force.

^a^ The 95% CIs were based on Monte Carlo simulation with 10 000 iterations.

### Cost-Effectiveness Analysis

We assessed the cost-effectiveness and mortality benefits of a non–risk-based, age-based LDCT lung cancer screening program for adults aged 40 to 85 years at current 14.4% and a hypothetical 70% participation (comparable to breast and colorectal rates of 67% and 69%, respectively) (Table 4). At 14.4% participation, such a lung screening program would cost $2.1 billion annually (95% CI, $1.5-$2.8 billion), preventing 20 500 lung cancer deaths (95% CI, 12 000-29 000 deaths) at a 30% stage I detection rate. This yielded a cost of $101 000 per life saved (95% CI, $82 000-$120 000 per life saved) and an ICER of $85 000 per QALY (95% CI, $60 000-$120 000 per QALY). Including a 6.7% all-cause mortality reduction as demonstrated in the prior studies, it would save 103 000 lives (95% CI, 70 000-140 000 lives) at $20 400 per life saved (95% CI, $14 000-$30 000 per life saved). At 70% participation, lung screening would cost $12.8 billion annually (95% CI, $8.8-$16.8 billion), preventing 99 700 lung cancer deaths (95% CI, 55 300-144 100 deaths). The cost per life saved would increase to $128 000 (95% CI, $88 000-$188 000 per life saved), with an ICER of $107 000 per QALY (95% CI, $73 000-$151 000 per QALY). With all-cause benefits, it would save 500 600 lives (95% CI, 339 900-661 300 lives) at $25 600 per life saved (95% CI, $17 500-$37 600 per life saved). The increased ICER and cost per life saved (approximately 26% higher) reflected higher screening volumes (64.1 vs 13.2 million annually) and fixed costs, yet the program remained cost-effective (<$150 000 per QALY). Recent data suggest that stage IV treatment with immunotherapy or targeted therapy costs $380 000 to $520 000 over 2 years, which is nearly 8 times the cost of stage I treatment ($53 000-$67 000).^23,24,25,26,27^ Hence, age-based screening achieving 30% stage I detection would save $24.76 billion annually, far exceeding program costs (eTable 7 in Supplement 1).

**Table 4.** Cost-Effectiveness of a Universal Lung Cancer Screening With Low-Dose Computed Tomography With 14.4% and 70% Participation Compared With Breast and Colon Cancer Screening

Metric	Mean (95% CI)			
	Lung (14.4% participation)^a^	Lung (70% participation)	Breast (67% participation)	Colorectal (69% participation)
Annual cost, $ billion	2.1 (1.5-2.8)	12.8 (8.8-16.8)	9.5 (7.0-12.0)	12.6 (9.5-16.0)
Lives saved				
Cancer specific	20 500 (12 000-29 000)	99 700 (55 300-144 100)	10 700 (8700-12 700)	13 700 (11 000-16 500)
All cause^b^	103 000 (70 000-140 000)	500 600 (339 900-661 300)	NA	NA
Cost per life saved, $1000				
Cancer specific	102 (70-150)	128 (88-188)	890 (700-1100)	920 (700-1200)
All cause	20.4 (14-30)	25.6 (17.5-37.6)	NA	NA
ICER/QALY, $1000^c^	85 (60-120)	107 (73-151)	50 (40-60)	25 (18-35)

Abbreviations: ICER, incremental cost-effectiveness ratio; NA, not applicable; QALY, quality-adjusted life-year.

^a^ Lung screening assumes 30% stage I detection (vs 16% baseline).

^b^ All-cause mortality includes 6.7% reduction for lung screening.

^c^ QALYs discounted at 3% annually, with mean (SD) weights of 0.85 (0.05).

In comparison, breast cancer screening in the simulation cost $9.5 billion annually (95% CI, $7.0-$12.0 billion), prevented 10 700 deaths (95% CI, 8700-12 700 deaths), with a cost of $890 000 per life saved (95% CI, $700 000-$1 100 000 per life saved) and ICER of $50 000 per QALY (95% CI, $40 000-$60 000 per QALY). Colorectal screening cost $12.6 billion (95% CI, $9.5-$16.0 billion), prevented 13 700 deaths (95% CI, 11 000-16 500 deaths), with a cost of $920 000 per life saved (95% CI, $700 000-$1 200 000 per life saved) and ICER of $25 000 per QALY (95% CI, $18 000-$35 000 per QALY). Hence, lung screening cost per life saved was 6.9 to 7.6 times lower than breast and colorectal at both participation rates, and 30 to 45 times lower with all-cause benefits, although its ICER was higher due to shorter survival and lower QALY weights (Table 4). Sensitivity analysis confirmed robust findings across all parameter ranges, with 98.7% probability of superior cost-effectiveness vs breast screening and 99.1% vs colorectal screening. The program would achieve cost-neutrality at 76% participation through treatment savings alone.

### Modeling of Risks Associated With Procedures to Diagnose Benign Disease

At 14.4% participation, lung screening would generate 3.3 million false-positive results annually (95% CI, 2.4-4.3 million results), or 14.7% per person screened (95% CI, 10.7%-19.2% per person screened), if the output is lung cancer diagnosis. This could lead to 260 000 invasive procedures (95% CI, 150 000-400 000 procedures), or 1.2% per person (95% CI, 0.7%-1.8% per person), with 1000 complications (95% CI, 500-1800 complications), or 0.004% per person (95% CI, 0.002%-0.008% per person). At 70% participation, false-positive results scaled to 16.0 million (95% CI, 11.1-20.9 million results), procedures to 1.26 million (95% CI, 0.73-1.94 million procedures), and complications to 4900 (95% CI, 2400-8700 complications), with unchanged per-person risks. Breast screening produced 3.3 million false-positive results (95% CI, 2.3-4.3 million results), or 7.5% per person, with 33 000 biopsies (95% CI, 16 000-65 000 biopsies), or 0.08% per person, and 500 complications (95% CI, 200-1000 complications), or 0.001% per person. Colorectal screening yielded 2.1 million false-positive results (95% CI, 1.5-3.2 million results), or 2.9% per person, with 420 000 polypectomies (95% CI, 270 000-640 000 polypectomies), or 0.58% per person, and 3200 complications (95% CI, 1800-4800 complications), or 0.004% per person (eTable 8 in Supplement 1).

### Radiation Risk Assessment

Under a risk-agnostic universal screening protocol, individuals screened every 10 years from ages 40 to 85 years would receive 5 LDCT scans totaling 6.5 mSv. The 15% requiring annual surveillance would accumulate 26 to 59 mSv (eTable 9 in Supplement 1). Lifetime radiation-induced cancer risk varies by age and sex. Starting at age 40 years, estimated risk was 0.04% for men and 0.05% for women, decreasing with later initiation because of latency periods and competing mortality (eTable 10 in Supplement 1). The benefit-to-risk ratio favored screening across all groups. For every radiation-induced cancer death, 108 to 284 lung cancer deaths would be prevented, depending on screening initiation age (eTable 11 in Supplement 1). Among 155.5 million eligible adults, we projected 435 radiation-induced cancer deaths vs 783 720 lung cancer deaths prevented over 30 years, or 1 radiation-induced cancer per 1800 lung cancer deaths prevented (eTable 12 in Supplement 1).

## Discussion

In this cohort study, 64.9% of patients with lung cancer did not meet USPSTF criteria, indicating that current guidelines would miss most cases even with perfect participation. The nonguideline group, enriched with women, Asian patients, never-smokers, and long-term former smokers, demonstrated superior OS, suggesting that these patients would benefit from systematic early detection. Expanding criteria to ages 40 to 85 years, at least 10 pack-years, and no smoking cessation limit would increase detection from 35.1% to 62.1%, yet 38.0% of patients, predominantly never-smokers, would remain ineligible, highlighting persistent gaps in addressing nontobacco risks and health inequities.^14,15,16,17^

Unlike risk-based lung cancer screening focused exclusively on tobacco smoking, universal breast and colorectal cancer programs have simplified access and increased participation.^28^ Risk-specific guidelines may deter participation by implying lifestyle blame or creating eligibility confusion, compounded by stigma.^29^ The National Lung Screening Trial,^3^ Early Lung Cancer Action Project,^30^ and NELSON (Dutch-Belgian Randomized Lung Cancer Screening Trial)^31^ confirmed LDCT’s mortality benefits. Our LDCT-screened patients showed earlier-stage diagnoses and improved survival, demonstrating broader screening potential. Economic modeling revealed universal screening would cost $101 000 per life saved, substantially outperforming breast ($890 000) and colorectal ($920 000) screening. Stage-specific treatment costs highlighted this disparity: stage IV treatment with modern immunotherapy or targeted therapy costs $380 000 to $520 000, vs $53 000 to $67 000 for stage I treatment.^32^ Age-based screening achieving 30% stage I detection would save $24.76 billion annually in treatment costs alone, far exceeding the $2.6 billion screening program cost.

Sensitivity analyses confirmed robust findings across all parameter ranges. Even under worst-case assumptions (20% stage I detection, 10% participation), universal screening remained more cost-effective than current breast or colorectal programs. Monte Carlo simulations demonstrated 98.7% probability of superior cost-effectiveness vs breast screening and 99.1% vs colorectal screening. The program would achieve cost-neutrality at 76% participation through treatment savings alone.

The paradoxical finding of higher stage I prevalence in the nonguideline group (28.0% vs 21.0%) likely reflects differences in tumor biology and detection patterns. The nonguideline group’s predominance of adenocarcinoma potentially represents relatively slower-growing tumors that are more likely to be detected incidentally before symptom development, contrasting with the guideline group’s more aggressive histologic profiles, including small cell carcinomas. Importantly, despite eligibility, only 12.6% of guideline patients underwent LDCT screening, with most presenting symptomatically with advanced disease. This observation strengthens our argument that early detection even when incidental improves outcomes.

Although approximately 25% of initial LDCT scans identified nodules requiring follow-up, contemporary protocols ensure that more than 92% are managed with imaging.^3,30,33^ False-positive rates exceeded mammography but remained manageable, with complication rates comparable to those for colonoscopy screening.^32^ Advances in liquid biopsies and minimally invasive procedures could further reduce false positives.^34,35,36,37^ Radiation exposure posed minimal risk, with 108 to 284 lung cancer deaths prevented per radiation-induced cancer death, maintaining favorable benefit-to-risk ratios even under worst-case assumptions.^38,39^

Beyond lung cancer detection, LDCT has been found to identify clinically important incidental findings in 50% to 60% of participants and to be effective in reducing all-cause mortality.^3,40^ The National Lung Screening Trial reported coronary artery calcification in 39% of participants enabling cardiovascular risk stratification,^41^ emphysema in 24%, and other malignant entities in 1.7%.^40^ The NELSON trial demonstrated coronary artery calcification detection alone reduced cardiovascular mortality by 12% through preventive interventions.^42,43^ LDCT also identifies aortic aneurysms (1.9%), osteoporotic fractures (8%-14%), and other actionable findings.^42,43^ The Early Lung Cancer Action Project estimated that these findings prevented 1 cardiovascular death per 7 lung cancer deaths prevented.^44^ The Multi-Ethnic Study of Atherosclerosis estimated coronary artery calcification screening alone provides $1000 to $2500 per person in health care savings.^45^ Thus, LDCT functions as comprehensive chest health assessment, substantially enhancing its value proposition.

Despite promise, implementation barriers persist since awareness remains low, rural access is limited, and stigma is prevalent, with uptake less than 15% vs 60% to 80% for breast or colorectal screening.^5,36^ Simplifying eligibility criteria could improve participation, although clinician education remains essential.^37^

### Limitations

This study has limitations that should be mentioned. The single-institution analysis may not reflect national demographics, limiting generalizability. The 70% participation assumption for expanded screening exceeds current rates, although sensitivity analyses demonstrated consistent benefits across all participation levels. Treatment cost estimates may prove conservative given emerging therapies. Breast and colorectal cost-effectiveness benchmarks may be overestimated, although our comprehensive analysis supports the comparison.

## Conclusions

Current USPSTF guidelines missed nearly two-thirds of cases, disproportionately excluding women, individuals from minoritized racial and ethnic groups, and never-smokers with favorable prognoses. Age-based screening (40-85 years) could enhance detection to 93.9%, prevent 26 124 deaths annually, and prove 6-fold more cost-effective than existing cancer screening programs. Policy revisions should expand eligibility, address nontobacco risks, and mitigate implementation barriers to ensure equitable early detection.

## Notes

Supplement 1. eAppendix. Supplemental Methods eTable 1. Initial Consulting Service for Lung Cancer Diagnosis by USPSTF Screening Eligibility eTable 2. Association Between Smoking Status at Diagnosis and Overall Survival in Lung Cancer Patients (Univariate Cox Proportional Hazards Model) eTable 3. Univariate Cox Proportional Hazards Analysis of Clinical and Pathological Factors Associated with Overall Survival eTable 4. Clinical Stage Distribution in LDCT-Screened vs. Non-Screened Patients Within the Guideline Group (n=350) (p<0.001) eTable 5. Proportions of patients that would remain ineligible for screening under the proposed modified screening guidelines eTable 6. Sensitivity Analysis for Main Table 3 Parameters eTable 7. Economic Impact of Stage Shift Through Universal Age-Based Lung Cancer Screening eTable 8. Modeling of Risks Associated With Procedures to Diagnose Benign Disease eTable 9. Cumulative Lifetime Radiation Exposure by Screening Protocol eTable 10. Estimated Radiation-Induced Cancer Risk by Age and Sex eTable 11. Benefit-to-Risk Ratios by Age and Sex - Radiation eTable 12. Population-Level Radiation Impact Over 30-Year Program

Supplement 2. Data Sharing Statement
