# Supplementary material for: Age-Based Screening for Lung Cancer Surveillance in the US
Source: JAMA Netw Open. 2025 Nov 20;8(11):e2546222. doi: 10.1001/jamanetworkopen.2025.46222 (PMC12635884; doi:10.1001/jamanetworkopen.2025.46222)
Supplement: Supplement 1. — eAppendix. Supplemental Methods eTable 1. Initial Consulting Service for Lung Cancer Diagnosis by USPSTF Screening Eligibility eTable 2. Association Between Smoking Status at Diagnosis and Overall Survival in Lung Cancer Patients (Univariate Cox Proportional Hazards Model) eTable 3. Univariate Cox Proportional Hazards Analysis of Clinical and Pathological Factors Associated with Overall Survival eTable 4. Clinical Stage Distribution in LDCT-Screened vs. Non-Screened Patients Within the Guideline Group (n=350) (p<0.001) eTable 5. Proportions of patients that would remain ineligible for screening under the proposed modified screening guidelines eTable 6. Sensitivity Analysis for Main Table 3 Parameters eTable 7. Economic Impact of Stage Shift Through Universal Age-Based Lung Cancer Screening eTable 8. Modeling of Risks Associated With Procedures to Diagnose Benign Disease eTable 9. Cumulative Lifetime Radiation Exposure by Screening Protocol eTable 10. Estimated Radiation-Induced Cancer Risk by Age and Sex eTable 11. Benefit-to-Risk Ratios by Age and Sex - Radiation eTable 12. Population-Level Radiation Impact Over 30-Year Program [file jamanetwopen-e2546222-s001.pdf]

Supplemental Online Content

Yang HC, Chang A, Visa M, et al. Age-based screening for lung cancer surveillance in the US. *JAMA Netw Open*. 2025;8(11):e2546222.
doi:10.1001/jamanetworkopen.2025.46222

eAppendix. Supplemental Methods

eTable 1. Initial Consulting Service for Lung Cancer Diagnosis by USPSTF Screening Eligibility

eTable 2. Association Between Smoking Status at Diagnosis and Overall Survival in Lung Cancer Patients (Univariate Cox Proportional Hazards Model)

eTable 3. Univariate Cox Proportional Hazards Analysis of Clinical and Pathological Factors Associated with Overall Survival

eTable 4. Clinical Stage Distribution in LDCT-Screened vs. Non-Screened Patients Within the Guideline Group (n=350) (p<0.001)

eTable 5. Proportions of patients that would remain ineligible for screening under the proposed modified screening guidelines.

eTable 6. Sensitivity Analysis for Main Table 3 Parameters

eTable 7. Economic Impact of Stage Shift Through Universal Age-Based Lung Cancer Screening

eTable 8. Modeling of Risks Associated With Procedures to Diagnose Benign Disease

eTable 9. Cumulative Lifetime Radiation Exposure by Screening Protocol

eTable 10. Estimated Radiation-Induced Cancer Risk by Age and Sex

eTable 11. Benefit-to-Risk Ratios by Age and Sex

eTable 12. Population-Level Radiation Impact Over 30-Year Program

eReferences

This supplemental material has been provided by the authors to give readers additional information about their work.

© 2025 Yang HC et al. *JAMA Network Open*.

eAppendix. Supplemental Methods

Study Design and Population

We conducted an institutional review board (IRB)–approved (No: STU00220033) retrospective cohort study at a large academic medical center, analyzing 997 consecutive patients with confirmed lung cancer. To minimize selection bias, patients were identified starting March 20, 2023, and selected sequentially backward in time. Patients were stratified by U.S. Preventive Services Task Force (USPSTF) 2021 screening eligibility into the Guideline Group (aged 50–80 years, ≥20 pack-year smoking history, current smokers or quit within 15 years) and the Non-Guideline Group (all others).

Data Collection

Data were extracted from electronic medical records. Demographic data included age at diagnosis, sex, race, and ethnicity. Smoking history comprised pack-years, smoking status (current, former, never), and years since cessation for former smokers. Clinical presentation data included symptoms at diagnosis (e.g., hemoptysis, dyspnea), referral source (primary care, emergency department, specialist), and imaging indication (symptomatic vs. incidental). Additionally, pretreatment pulmonary function tests (forced expiratory volume in 1 second [FEV1] and diffusing capacity for carbon monoxide [DLCO] as percent predicted), tumor characteristics (histology, American Joint Committee on Cancer [AJCC] stage at diagnosis), and survival outcomes (time from diagnosis to death or last follow-up) were recorded.

Statistical Analysis

Continuous variables were summarized using means with standard deviations for normally distributed data or medians with interquartile ranges for skewed distributions. Categorical variables were presented as frequencies and percentages. Group comparisons employed chi-square tests for categorical variables and Wilcoxon rank-sum tests for continuous variables.

Survival analysis utilized Kaplan-Meier curves with log-rank tests to compare overall survival between Guideline and Non-Guideline groups. We constructed univariate Cox proportional hazards models to evaluate associations between overall survival and key variables including guideline group status, sex, smoking status, tumor stage, and histology. All analyses were performed using R version 4.3.1 with packages including 'survival' for survival analysis, 'gtsummary' for descriptive statistics, and 'ggplot2' for visualizations. Two independent biostatisticians conducted all statistical analyses to ensure accuracy.

Expanded Screening Criteria Analysis

We modeled the impact of expanded eligibility criteria through hypothetical scenarios, including extending the age range to 40–85 years, lowering the smoking threshold to ≥10 pack-years, including all ever-smokers regardless of quit duration, and combinations thereof. Detection rates were calculated as the proportion of our cohort captured under each scenario. To contextualize findings, we compared lung cancer screening uptake with national breast and colorectal screening data. Population estimates used 2024 U.S. Census Bureau projections: 155.5 million adults aged 40–85 for lung, 65 million women aged 40–74 for breast, and 105 million adults aged 45–75 for colorectal screening. Disease parameters from the Surveillance, Epidemiology, and End Results (SEER) program included annual incidence (237,197 for lung, 297,790 for breast, 153,020 for colorectal) and mortality (127,070, 43,170, 52,550, respectively).[1-4]

© 2025 Yang HC et al. *JAMA Network Open*.

Mortality Impact Modeling

We modeled the mortality impact of shifting lung cancer diagnoses from late to early stages using stage-specific mortality rates from SEER, validated by our cohort. Each 1% shift from Stage IV to Stage I detection was estimated to shift 2,372 cases (1% of annual lung cancer incidence) and prevent 1,874 deaths, based on mortality differences between Stage IV (94%) and Stage I (15%). To benchmark effectiveness, we calculated the Stage I detection rate required for universal lung screening to match lives saved by breast (10,660 annually, 8.2 deaths averted per 1,000 women screened over a lifetime) and colorectal (13,650 annually, 26 deaths averted per 1,000 adults screened from ages 45–75) screening.[1-4]

Assumptions for Screening Programs

We developed a comparative cost-effectiveness model from a health care system perspective with a lifetime horizon, evaluating three cancer screening strategies: universal LDCT lung screening for adults aged 40–85 years regardless of smoking history, biennial mammography for women aged 40–74 years, and colonoscopy every 10 years for adults aged 45–75 years. Population estimates were derived from 2024 U.S. Census projections. SEER data provided incidence and mortality rates, and stage-specific 5-year survival rates for lung cancer (Stage IA: 92%, Stage IB: 83%, Stage IIA: 65%, Stage IIB: 53%, Stage III: 25%, Stage IV: 6%) were validated by our cohort. Current lung cancer stage distribution was 16% Stage I, 8% Stage II, 26% Stage III, and 50% Stage IV. Breast and colorectal screening effectiveness data were sourced from USPSTF-commissioned studies.[2,3]

Cost-Effectiveness Analysis

Cost-effectiveness was assessed using Monte Carlo simulations in Python (numpy, scipy), modeling lung screening at 14.4% (real-world uptake) and 70% participation (comparable to breast and colorectal rates of 67% and 69%) to evaluate scalability. Data were sourced from our cohort, NLST for mortality reductions (20% lung cancer, 6.7% all-cause), USPSTF reports for parameters, SEER for survival, CDC for all-cause mortality (1,000/100,000 annually for ages 40–85), and 2024 Medicare rates ($200/LDCT, $290/mammogram, $1,195/colonoscopy). Simulations used a health care perspective, lifetime horizon, and 3% annual discount for costs and QALYs, running 10,000 iterations. For lung screening, we assumed 155.5 million eligible adults, LDCT every 10 years with 15% annual surveillance, beta-distributed participation (14.4%: α=2, β=10; 70%: α=35, β=15), yielding 13.2 million (95% CI: 9.1–17.3 million) and 64.1 million (95% CI: 44.7–83.5 million) screenings. LDCT cost was $200 (SD $50), with 20,500 lung cancer lives saved (95% CI: 12,000–29,000) at 14.4%, scaled to 99,700 (95% CI: 55,300–144,100) at 70%, based on 30% Stage I detection. All-cause reduction saved 103,000 lives (95% CI: 70,000–140,000) at 14.4%, scaled to 500,600 (95% CI: 339,900–661,300) at 70%. QALYs used a weight of 0.85 (SD 0.05) with 10-year survival. Breast screening assumed 65 million women, 67% participation, 32.5 million screenings (95% CI: 22.5–42.5 million), $290/mammogram (SD $72.5), 10,700 lives saved (95% CI: 8,700–12,700), and QALY weight of 0.85 with 15-year survival. Colorectal screening assumed 105 million adults, 69% participation, 10.5 million screenings (95% CI: 6.5–14.5 million), $1,195/colonoscopy (SD $298.75), 13,700 lives saved (95% CI: 11,000–16,500), and QALY weight of 0.85 with 15-year survival. We assumed linear scaling of lives saved, fixed costs, and no economies of scale.

We also performed a Monte Carlo simulation based on CISNET-style modeling to compare LDCT lung cancer screening with breast and colorectal cancer screening. Parameters were derived from national data and our cohort analysis. Simulations ran 10,000 iterations per

© 2025 Yang HC et al. *JAMA Network Open*.

strategy using normally distributed assumptions for screening cost, QALYs gained, and lives saved. Costs were based on Medicare estimates ($200 per LDCT, $290 per mammogram, $1,195 per colonoscopy). QALYs per life saved were modeled at 0.85 (SD 0.05) for all cancer types, and outcomes were discounted at 3% annually. The incremental cost-effectiveness ratio (ICER) and cost per life saved were computed across simulations, with results summarized as means and 95% confidence intervals.

Modeling of Risks Associated With Procedures to Diagnose Benign Disease

Testing performed to diagnose non-malignant findings was modeled to estimate false-positive results, invasive procedures (biopsies for lung and breast, polypectomies for colorectal), and complications. For lung screening, we assumed a false-positive rate of 25% (triangular distribution, range 20–30%), an invasive procedure rate of 8% (range 5–10%, 92% non-invasive management), and a complication rate of 0.4% (range 0.2–0.6%, e.g., pneumothorax). Breast screening used a false-positive rate of 10% (range 7–12%), procedure rate of 1% (range 0.5–2%), and complication rate of 1.5% (range 1–2%, e.g., infection). Colorectal screening assumed a false-positive rate of 20% (range 15–30%), procedure rate of 20% (range 15–25%), and complication rate of 0.75% (range 0.5–1%, e.g., perforation). Absolute outcomes scaled with screening volume, while per-person risks remained constant, based on NLST and literature. Outcomes included annual counts and per-person percentages.

Number Needed to Screen (NNS)

NNS was calculated as the number of annual screenings divided by lives saved, based on simulation means and CIs, assuming a constant NNS across participation rates due to proportional scaling.[2]

Stage-Shift Feasibility

Stage-shift feasibility was assessed by calculating Stage I detection rates needed to match breast (10,700 lives saved) and colorectal (13,700 lives saved) mortality benefits, using stage-shift model (1% Stage IV to Stage I shift prevents 1,874 deaths). Targets of 21.4% (breast) and 22.3% (colorectal) were validated against our cohort's 28% Stage I detection in Non-Guideline patients, with outcomes scaled to 20,500 (14.4%) and 99,700 (70%) deaths prevented at 30% Stage I detection. We assumed a linear relationship between Stage I detection and mortality reduction.

Modeling of Radiation Risk

Radiation risks were modeled for lung and breast screening, with colorectal screening assumed to have no radiation exposure. Each LDCT delivered 1.3 mSv (SD 0.2 mSv), with 85% receiving 5 scans (6.5 mSv) and 15% receiving 20–45 scans (26–59 mSv) over a lifetime, per American College of Radiology standards. Breast screening used 0.4 mSv per mammogram (SD 0.05 mSv), with 17 exams (6.8 mSv). Cancer risk for lung was modeled with a triangular distribution (min 0.0003, mode 0.0005, max 0.0007 per mSv) and for breast at 1.5×10^{-5} per mSv (range $1–2 \times 10^{-5}$), using BEIR VII's linear no-threshold model, with fatality rates of 5.8% (SD 1%) for lung and 20% (range 15–25%) for breast. Lives saved scaled with participation (20,500 at 14.4%, 99,700 at 70% for lung; 10,700 for breast), and outcomes included cancers, deaths, and benefit-to-risk ratio (lives saved ÷ deaths). We assumed no interaction effects between scans.

Simulations and Assumptions

Simulations ran 10,000 iterations to estimate means and 95% CIs (2.5th–97.5th percentiles) for all outcomes, using beta distributions for participation, normal distributions for costs and lives saved, and triangular distributions for rates (false-positive, procedure, complication, cancer risk). Data were analyzed in Python 3.9 with numpy, scipy, and pandas. Key assumptions included linear scaling of benefits and risks, fixed costs, constant per-person risks, and no overdiagnosis, potentially overestimating benefits. Participation was based on real-world data (14.4% for lung) and achievable targets (70% for lung, 67% for breast, 69% for colorectal). Limitations include simplified cancer progression, fixed cost assumptions, and cohort generalizability.

Ethical Considerations

The study adhered to the Declaration of Helsinki. All patient data were deidentified before analysis to ensure confidentiality. The IRB reviewed and approved all procedures.

© 2025 Yang HC et al. *JAMA Network Open*.

eTable 1. Initial Consulting Service for Lung Cancer Diagnosis by USPSTF Screening Eligibility

First Consultant	Overall (n=997)	Guideline (n=350)	Non-Guideline (n=647)	P value
Emergency Department	320 (32.1%)	110 (31.4%)	210 (32.5%)	.70
Primary Care	280 (28.1%)	104 (29.7%)	176 (27.2%)	.40
Unknown	165 (16.5%)	60 (17.1%)	105 (16.2%)	.70
Other	83 (8.3%)	25 (7.1%)	58 (9.0%)	.30
Medical Oncology	63 (6.3%)	18 (5.1%)	45 (7.0%)	.30
Respiratory Medicine	59 (5.9%)	28 (8.0%)	31 (4.8%)	.04
Thoracic Surgery	27 (2.7%)	5 (1.4%)	22 (3.4%)	.07

© 2025 Yang HC et al. *JAMA Network Open*.

eTable 2. Association Between Smoking Status at Diagnosis and Overall Survival in Lung Cancer Patients (Univariate Cox Proportional Hazards Model)

Smoking status at diagnosis	N	Event number	HR	95% CI	P value
Current	166	84	—	—	<.001
Never	247	67	0.49	0.35, 0.67	<.001
Quit duration <=10yr	247	116	0.92	0.70, 1.22	.60
Quit duration 10-30yr	190	80	0.73	0.54, 0.99	.05
Quit duration >30 yr	147	54	0.65	0.46, 0.92	.02

© 2025 Yang HC et al. *JAMA Network Open*.

eTable 3. Univariate Cox Proportional Hazards Analysis of Clinical and Pathological Factors Associated with Overall Survival

Variables (N)	N	Event	HR	95% CI	P value
Cohort (997)					
Guideline	350	172	—	—	—
Non-Guideline	647	229	0.67	0.55, 0.82	<.001
Age at Diagnosis	997	401	1.01	1.00, 1.02	.02
Sex (997)					
Female	577	200	—	—	—
Male	420	201	1.57	1.29, 1.91	<.001
Stage (990)					<.001
1	251	47	—	—	
2	115	33	2.06	1.32, 3.22	.001
3	191	71	2.88	1.99, 4.18	<.001
4	433	247	5.97	4.35, 8.20	<.001
Histology (996)					<.001
ADC	661	247	—	—	—
Carcinoid	29	3	0.20	0.06, 0.61	.005
Large Cell	14	7	1.48	0.70, 3.14	.30
NSCLC, NOS	73	32	1.31	0.91, 1.91	.15
Sarcomatoid	3	1	1.26	0.18, 9.00	.80
SCLC	70	50	2.94	2.17, 4.00	<.001
SQC	146	60	1.27	0.95, 1.68	.10

ADC, Adenocarcinoma; NSCLC, Non-small cell lung cancer; NOS, Not Otherwise Specified; SCLC, Small cell lung cancer; SQC, Squamous cell carcinoma.

© 2025 Yang HC et al. *JAMA Network Open*.

eTable 4. Clinical Stage Distribution in LDCT-Screened vs. Non-Screened Patients Within the Guideline Group (n=350) (p<0.001)

Clinical Stage	LDCT (44)	No LDCT (306)
1	16 (36%)	60 (20%)
2	11 (25%)	32 (10%)
3	9 (20%)	72 (24%)
4	8 (18%)	142 (46%)

LDCT, Low-Dose Computed Tomography

© 2025 Yang HC et al. *JAMA Network Open*.

eTable 5. Proportions of patients that would remain ineligible for screening under the proposed modified screening guidelines.

Eligibility category	N (%)
Eligible under Current Guideline	350 (35%)
Additional Patients Eligible under the New Guideline	266 (27%)
Still ineligible despite the New Guideline	
1. Only age (<40 or >85)	23 (2.3%)
2-1 Only PY <10	93 (9.3%)
2-2 Never smoker	223 (22%)
2-3 Unknown PY	4 (0.4%)
3-1 Not in age range and PY <10 (smoker)	11 (1.1%)
3-2 Not in age range and PY <10 (never smoker)	26 (2.6%)
3-3 Not in age range and unknown PY (smoker)	1 (0.1%)

PY, pack-years

© 2025 Yang HC et al. *JAMA Network Open*.

eTable 6. Sensitivity Analysis for Main Table 3 Parameters

One-Way Sensitivity Analysis: Impact of Individual Parameter Changes

Parameter	Base Case Value	Range Tested	Impact on Lives Saved (30% Stage I)	Impact on Cost per Life Saved
Stage I detection rate	30%	20-40%	17,416-34,832	$148,000-$76,000
Mortality reduction per 1% stage shift	1,874 deaths	1,500-2,200	22,500-33,000	$113,000-$77,000
Screening participation rate	14.4%	10-70%	18,142-127,000	$116,000-$101,000
LDCT sensitivity	94%	85-99%	23,400-27,500	$112,000-$95,000
LDCT specificity	73%	60-85%	24,800-27,400	$106,000-$96,000
LDCT cost	$200	$150-$300	No change	$76,000-$151,000
False positive follow-up cost	$500	$300-$1000	No change	$95,000-$118,000
Stage I, 5-year survival	92%	85-95%	24,200-28,100	$109,000-$94,000
Stage IV, 5-year survival	6%	3-10%	28,500-23,700	$93,000-$111,000

Two-Way Sensitivity Analysis: Combined Parameter Variations

Scenario	Stage I Detection	Participation Rate	Lives Saved	Cost per Life Saved	Total Program Cost
Conservative	25%	10%	15,118 (12,000-18,236)	$139,000 ($115,000-$163,000)	$2.1B ($1.7B-$2.5B)
Base Case	30%	14.4%	26,124 (20,000-32,248)	$101,000 ($82,000-$120,000)	$2.6B ($2.1B-$3.1B)
Moderate	30%	40%	72,567 (58,000-87,134)	$105,000 ($87,000-$123,000)	$7.6B ($6.3B-$8.9B)

© 2025 Yang HC et al. *JAMA Network Open*.

Scenario	Stage I Detection	Participation Rate	Lives Saved	Cost per Life Saved	Total Program Cost
Optimistic	35%	70%	167,305 (134,000-200,610)	$88,000 ($73,000-$103,000)	$14.7B ($12.2B-$17.2B)

Threshold Analysis: Key Break-Even Points

Metric	Threshold Value	Interpretation
Stage I detection to match breast screening cost-effectiveness	8.2%	Any detection >8.2% is more cost-effective than breast
Stage I detection to match colorectal cost-effectiveness	8.5%	Any detection >8.5% is more cost-effective than colorectal
Participation rate for cost neutrality	76%	Program becomes cost-neutral at 76% participation due to treatment savings
Stage I detection for 50,000 lives saved	45%	Would need 45% Stage I detection to save 50,000 lives annually
Maximum acceptable LDCT cost	$894	LDCT could cost up to $894 and still match breast screening efficiency

Probabilistic Sensitivity Analysis (Monte Carlo Simulation Results)

Distribution Parameters Used:

- Stage I detection: Beta distribution (α=30, β=70)
- Cost per LDCT: Gamma distribution (shape=4, scale=50)
- Sensitivity/Specificity: Beta distributions
- Mortality benefit: Normal distribution (mean=1,874, SD=187)

© 2025 Yang HC et al. *JAMA Network Open*.

Results from 10,000 Iterations:

- Mean lives saved: 26,124
- 95% credible interval: 20,000-32,248
- Probability more cost-effective than breast screening: 98.7%
- Probability more cost-effective than colorectal screening: 99.1%
- Probability of saving >20,000 lives: 84.3%
- Probability cost per life saved <$150,000: 95.2%

Parameters with Greatest Impact on Cost-Effectiveness

Parameters ranked by impact on incremental cost-effectiveness ratio (ICER):

1. Stage I detection rate (±40% change in ICER)
2. LDCT cost (±35% change in ICER)
3. Mortality benefit per stage shift (±28% change in ICER)
4. False positive rate (±18% change in ICER)
5. Screening participation (±15% change in ICER)
6. LDCT sensitivity (±12% change in ICER)
7. Follow-up costs (±8% change in ICER)
8. LDCT specificity (±5% change in ICER)

Scenario Analysis: Best and Worst Cases

Scenario	Assumptions	Lives Saved	Cost per Life Saved	ICER ($/QALY)
Worst Case	20% Stage I, 10% participation, high costs	12,062	$278,000	$232,000
Base Case	30% Stage I, 14.4% participation	26,124	$101,000	$85,000
Best Case	40% Stage I, 70% participation, low costs	195,930	$52,000	$43,000

© 2025 Yang HC et al. *JAMA Network Open*.

Key Findings from Sensitivity Analysis:

1. **Robustness**: Results remain favorable across all reasonable parameter ranges
2. **Stage I Detection**: Most influential parameter; even modest improvements (>22%) exceed current screening programs
3. **Cost-Effectiveness**: Remains superior to breast/colorectal screening unless Stage I detection falls below 8.5%
4. **Break-Even**: Program becomes cost-saving at 76% participation due to reduced treatment costs
5. **Uncertainty**: 95% probability that universal screening is more cost-effective than current programs

*All monetary values in 2024 USD; QALY = Quality-adjusted life year; ICER = Incremental cost-effectiveness ratio

© 2025 Yang HC et al. *JAMA Network Open*.

eTable 7. Economic Impact of Stage Shift Through Universal Age-Based Lung Cancer Screening

Panel A: Stage-Specific Treatment Costs

Stage	Treatment Modality	First-Year Cost	Second-Year Cost	Total 2-Year Cost	5-Year Total Cost
**Stage I**					
IA	Lobectomy/Segmentectomy	$45,000-55,000	$8,000-12,000	$53,000-67,000	$75,000-85,000
IB	SBRT (if inoperable)	$35,000-42,000	$8,000-10,000	$43,000-52,000	$65,000-75,000
**Stage II**					
IIA/IIB	Surgery + Adjuvant CTx	$75,000-95,000	$15,000-25,000	$90,000-120,000	$135,000-165,000
**Stage III**					
IIIA	CCRT + Durvalumab	$165,000-195,000	$85,000-115,000	$250,000-310,000	$425,000-525,000
IIIB/IIIC	Chemoimmunotherapy	$185,000-225,000	$95,000-135,000	$280,000-360,000	$475,000-575,000
**Stage IV**					
Driver mutation+	Targeted therapy (Osimertinib, Alectinib)	$195,000-235,000	$185,000-225,000	$380,000-460,000	$750,000-950,000
PD-L1 ≥50%	Pembrolizumab monotherapy	$175,000-215,000	$165,000-205,000	$340,000-420,000	$650,000-850,000
PD-L1 <50%	CTx + Pembrolizumab	$225,000-275,000	$195,000-245,000	$420,000-520,000	$850,000-1,050,000

SBRT, Stereotactic Body Radiation Therapy; CTx, Chemotherapy; CCRT, Concurrent Chemoradiotherapy; PD-L1, Programmed Death-Ligand 1.

© 2025 Yang HC et al. *JAMA Network Open*.

Panel B: Current vs. Universal Screening Stage Distribution and Costs

Stage	Current Distribution	Current Annual Cases	Universal Screening Distribution*	Universal Screening Cases	Cases Shifted
Stage I	16%	37,952	30%	71,159	+33,207
Stage II	8%	18,976	12%	28,464	+9,488
Stage III	26%	61,671	28%	66,415	+4,744
Stage IV	50%	118,599	30%	71,159	-47,440
**Total**	100%	237,197	100%	237,197	0

*Based on 30% Stage I detection rate achieved in Non-Guideline group.

Panel C: Annual Treatment Cost Analysis

Cost Category	Current Stage Distribution	Universal Screening	Annual Savings
**Direct Treatment Costs**			
Stage I treatments	$2.28 billion	$4.27 billion	-$1.99 billion
Stage II treatments	$1.90 billion	$2.85 billion	-$0.95 billion
Stage III treatments	$17.27 billion	$18.60 billion	-$1.33 billion
Stage IV treatments	$53.37 billion	$32.02 billion	+$21.35 billion
**Subtotal Treatment**	**$74.82 billion**	**$57.74 billion**	**+$17.08 billion**
**Additional Costs/Savings**			
Reduced ICU admissions	---	---	+$1.85 billion
Reduced emergency presentations	---	---	+$0.92 billion
Palliative care reduction	---	---	+$1.24 billion
Productivity gains (working age)	---	---	+$3.67 billion

© 2025 Yang HC et al. *JAMA Network Open*.

Cost Category	Current Stage Distribution	Universal Screening	Annual Savings
**Total Annual Savings**			**$24.76 billion**

Panel D: 10-Year Projection with Universal Screening Implementation

Year	Screening Participation	Cumulative Lives Saved	Cumulative Cost Savings	Net Program Value†
Year 1	14.4%	26,124	$3.57 billion	$1.47 billion
Year 2	20%	62,374	$8.49 billion	$4.29 billion
Year 3	30%	112,874	$16.84 billion	$9.84 billion
Year 5	45%	243,374	$41.21 billion	$26.71 billion
Year 10	70%	687,374	$142.85 billion	$102.85 billion

†Net value = Cost savings - Screening program costs

Key Economic Findings:

1. **Modern Treatment Cost Burden**: Stage IV treatment with immunotherapy/targeted therapy now costs $420,000-520,000 over 2 years, representing a 108% increase from 2015 costs
2. **Stage Shift Economics**: Each 1% shift from Stage IV to Stage I saves approximately $370,000 per patient in direct treatment costs
3. **Break-Even Analysis**: Universal screening becomes cost-neutral at 35% participation when treatment savings offset screening costs
4. **Immunotherapy Impact**: The availability of durvalumab for Stage III and pembrolizumab for Stage IV has increased treatment costs by 65-85% since 2018
5. **Targeted Therapy Burden**: Driver mutation-positive patients (15-20% of Stage IV) incur the highest costs at $750,000-950,000 over 5 years

© 2025 Yang HC et al. *JAMA Network Open*.

Methodology Notes:

- Costs derived from 2024 Medicare claims data and published cost-effectiveness analyses
- Includes direct medical costs only; indirect costs (lost productivity, caregiver burden) would increase savings
- Stage shift assumptions based on manuscript's observed 30% Stage I detection in Non-Guideline group
- All costs inflation-adjusted to 2024 USD using medical care Consumer Price Index (CPI)

© 2025 Yang HC et al. *JAMA Network Open*.

eTable 8. Modeling of Risks Associated With Procedures to Diagnose Benign Disease

Metric	Lung (14.4% Participation)	Lung (70% Participation)	Breast (67% Participation)	Colorectal (69% Participation)
**False Positives (Million)**	3.3 (2.4–4.3)	16.0 (11.1–20.9)	3.3 (2.3–4.3)	2.1 (1.5–3.2)
**Invasive Procedures (K)**	260 (150–400)	1,260 (730–1,940)	33 (16–65)	420 (270–640)
**Complications**	1,000 (500–1,800)	4,900 (2,400–8,700)	500 (200–1,000)	3,200 (1,800–4,800)
**False Positive per Person (%)**	14.7 (10.7–19.2)	14.7 (10.7–19.2)	7.5 (5.3–9.8)	2.9 (2.1–4.4)
**Procedure per person (%)**	1.2 (0.7–1.8)	1.2 (0.7–1.8)	0.08 (0.04–0.15)	0.58 (0.37–0.88)
**Complication per Person (%)**	0.004 (0.002–0.008)	0.004 (0.002–0.008)	0.001 (0.0005–0.002)	0.004 (0.002-0.007)

Values are means with 95% confidence intervals in parentheses; False positives, procedures, and complications scale with screening volume (13.2M at 14.4%, 64.1M at 70% for lung); Per-person risks remain unchanged, as they depend on individual screening outcomes; Breast and colorectal metrics are unchanged, as participation (67%, 69%) is near 70%; Lung procedures include biopsies; breast, biopsies; colorectal, polypectomies.

© 2025 Yang HC et al. *JAMA Network Open*.

eTable 9. Cumulative Lifetime Radiation Exposure by Screening Protocol

Screening Protocol	Number of Scans	Cumulative Dose (mSv)	Equivalent Background Years	Population Affected
Standard (every 10 years)	5	6.5	2.2	85%
Enhanced (every 5 years)	9	11.7	3.9	High-risk subset
Surveillance (annual)	20-45	26-59	8.7-19.7	15%
Weighted population average	6.8	8.8	2.9	100%

© 2025 Yang HC et al. *JAMA Network Open*.

eTable 10. Estimated Radiation-Induced Cancer Risk by Age and Sex

Age at First Screen	Male Risk (%)	Female Risk (%)	Combined Risk (%)	Expected Cancers per 100,000 Screened
40 years	0.040	0.055	0.048	48
50 years	0.032	0.044	0.038	38
60 years	0.024	0.033	0.029	29
70 years	0.016	0.022	0.019	19

© 2025 Yang HC et al. *JAMA Network Open*.

eTable 11. Benefit-to-Risk Ratios by Age and Sex

Age Group	Lung Cancer Deaths Prevented per 100,000	Radiation Deaths per 100,000	Benefit-to-Risk Ratio
40-49	3,150	12	263:1
50-59	5,400	19	284:1
60-69	8,200	31	265:1
70-79	9,800	52	188:1
80-85	7,500	69	108:1
Overall	6,800	28	243:1

© 2025 Yang HC et al. *JAMA Network Open*.

eTable 12. Population-Level Radiation Impact Over 30-Year Program

Outcome	Number	Rate per Million Screened
Lung cancers detected	7,115,910	45,780
Lung cancer deaths prevented	783,720	5,040
Radiation-induced cancers	7,464	48
Radiation-induced cancer deaths	435	2.8
Net lives saved	783,285	5,037

© 2025 Yang HC et al. *JAMA Network Open*.

eReferences

1. National Lung Screening Trial Research T, Aberle DR, Adams AM, et al. Reduced lung-cancer mortality with low-dose computed tomographic screening. *N Engl J Med*. Aug 4 2011;365(5):395–409. doi:10.1056/NEJMoa1102873

2. Siu AL, Force USPST. Screening for Breast Cancer: U.S. Preventive Services Task Force Recommendation Statement. *Ann Intern Med*. Feb 16 2016;164(4):279–96. doi:10.7326/M15-2886

3. Lin JS, Perdue LA, Henrikson NB, Bean SI, Blasi PR. Screening for Colorectal Cancer: Updated Evidence Report and Systematic Review for the US Preventive Services Task Force. *JAMA*. May 18 2021;325(19):1978–1998. doi:10.1001/jama.2021.4417

4. Toumazis I, de Nijs K, Cao P, et al. Cost-effectiveness Evaluation of the 2021 US Preventive Services Task Force Recommendation for Lung Cancer Screening. *JAMA Oncol*. Dec 1 2021;7(12):1833–1842. doi:10.1001/jamaoncol.2021.4942

© 2025 Yang HC et al. *JAMA Network Open*.
