# Supplementary material for: Age-Based Screening for Lung Cancer Surveillance in the US
Source: JAMA Netw Open. 2025 Nov 20;8(11):e2546222. doi: 10.1001/jamanetworkopen.2025.46222 (PMC12635884; doi:10.1001/jamanetworkopen.2025.46222)
Supplement: Supplement 2. — Data Sharing Statement [file jamanetwopen-e2546222-s002.pdf]

Data Sharing Statement

Yang. Age-Based Screening for Lung Cancer Surveillance in the US. *JAMA Netw Open*. Published November 20, 2025. doi:10.1001/jamanetworkopen.2025.46222

Data
Data available: No
